# Microbial Fermentation of Dietary Protein: An Important Factor in Diet–Microbe–Host Interaction

**DOI:** 10.3390/microorganisms7010019

**Published:** 2019-01-13

**Authors:** Natalie E. Diether, Benjamin P. Willing

**Affiliations:** Department of Agriculture, Food & Nutritional Science, University of Alberta, Ag/For Centre, Edmonton Alberta, T6G 2P5, Canada; nmay@ualberta.ca

**Keywords:** gut microbiota, protein fermentation, amino acids, host-microbial interaction

## Abstract

Protein fermentation by gut microbiota contributes significantly to the metabolite pool in the large intestine and may contribute to host amino acid balance. However, we have a limited understanding of the role that proteolytic metabolites have, both in the gut and in systemic circulation. A review of recent studies paired with findings from previous culture-based experiments suggests an important role for microbial protein fermentation in altering the gut microbiota and generating a diverse range of bioactive molecules which exert wide-ranging host effects. These metabolic products have been shown to increase inflammatory response, tissue permeability, and colitis severity in the gut. They are also implicated in the development of metabolic disease, including obesity, diabetes, and non-alcoholic fatty liver disease (NAFLD). Specific products of proteolytic fermentation such as hydrogen sulfide, ammonia, and *p*-Cresol may also contribute to the development of colorectal cancer. These findings are in conflict with other studies showing that tryptophan metabolites may improve gut barrier function and attenuate severity in a multiple sclerosis model. Further research examining proteolytic fermentation in the gut may be key to our understanding of how microbial and host metabolism interact affecting health.

## 1. Introduction

The gut microbiome is composed of a diverse range of species, the majority of which have a mutualistic relationship with the host, and, with 100 times more genes, perform metabolic functions greatly beyond those encoded in the host genome [1]. These reactions create secondary metabolites that can be beneficial or harmful. The ability of microbes to extract energy from indigestible carbohydrates and produce beneficial short-chain fatty acids (SCFAs) is well established [2,3]. Microbial metabolites are also increasingly recognized for their importance in modulating host metabolic and immune responses [3,4]. Much less is known about the role that proteolytic fermentation by gut microbes plays in host health and metabolism. However, it is clear that long-term dietary patterns, such as increased consumption of protein or decreased consumption of fiber, can shift the composition of the microbiota, changing which taxa and fermentation pathways are most abundant [5]. These system-level shifts in composition and metabolism hint at the complexity of metabolic interactions occurring in the gut microbial ecosystem, but more information is needed on the processes underlying these changes. Despite the efficiency of host digestion and absorption, some nitrogen-containing compounds in the intestine are metabolized by the microbiota. This is due both to the dispersal limitation of brush-border enzymes in the small intestine and excessive protein intake [6]. In the case of high-protein weight loss diets, protein intake may be 2–5 times greater than the daily dietary recommendations [7]. Understanding the fate of undigested dietary protein is an important consideration in determining the effects of long-term dietary patterns on health.

Amino acids are building blocks for microbial protein, making them important for microbial growth. However, they can also be fermented as an energy source [8]. Undigested peptides are broken down by proteolytic bacteria and subsequently used either in proteolytic fermentation or to form microbial cell components. Microbial protein has a high proportion of branched-chain amino acids, though the exact composition varies between bacterial strains [8]. Catabolism results in many metabolites that affect the host beyond amino acid availability. The fate of amino acids depends on ecological and dietary factors that influence the relative amounts of proteolytic fermentation. For example, low dietary fiber may result in increased proteolytic fermentation due to the low amount of fermentable carbohydrate in the colon [9]. Shifts toward increased proteolytic fermentation can alter the relative abundance of microbial species in the gut and generate bioactive, potentially deleterious metabolic products [8].

Limited information is available about the role of proteolytic fermentation in the complex metabolic networks between gut microbes and their host. More information is needed regarding what products are generated, which species are involved or affected, and how these changes come together to affect the host. Complementing other reviews, we will describe how pathways of proteolytic fermentation, metabolites produced, and dietary pattern converge to affect health. In particular, this review will focus on the compartment-specific effects of proteolytic fermentation in different segments of the intestine along with metabolites such as ammonia, p-cresol, and amines that may shape health.

## 2. Proteolytic Fermentation Involves Many Metabolic Pathways

**Figure 1:**
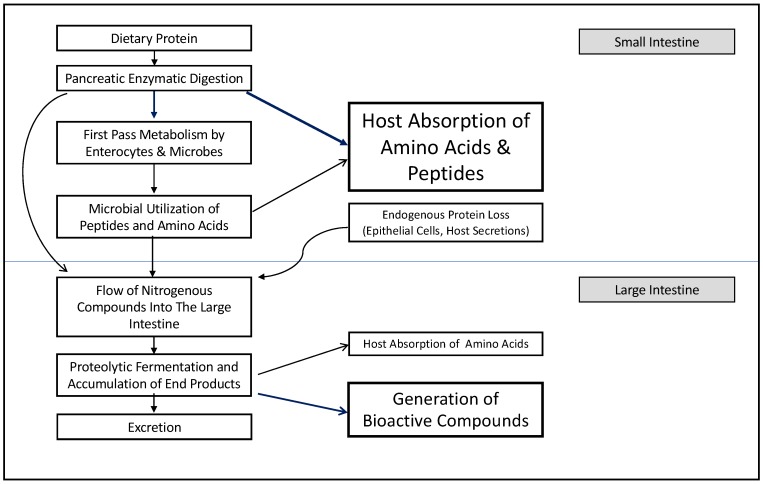
The fate of dietary protein in the gut is determined by a network of metabolic processes including both host and microbial digestion and utilization. The dominant effects for each compartment are bolded and shown with dark blue arrows.

The modeling of host and microbiota metabolic networks using genome annotation has identified 3499 distinct reactions; of these, 1267 are unique to the microbiota and 1142 are shared with the host [4]. When these reactions are attributed to larger functions, the intricate interdependencies of host and microbiota metabolism become evident. Three-quarters of all pathways utilize both host and microbiota reactions [4]. This collaboration of host and microbial reactions determines the fate of dietary protein in the gut, and the overall effects on host amino acid balance and metabolism (Figure 1). The interrelatedness of metabolic reactions also complicates the modeling of fermentation dynamics in the gut environment, limiting our current understanding of protein fermentation.

A protein that escapes host enzymatic digestion in the small intestine can be hydrolyzed by bacteria using extracellular proteases and peptidases resulting in free amino acids and peptides that can be taken up by the bacteria [10,11]. Culture-based experiments suggest that gut bacteria preferentially assimilate and ferment peptides over amino acids, a process which is more energetically efficient [6]. Once in the cytoplasm, amino acids can be incorporated into the microbial protein, or they can enter a catabolic pathway involving highly specific enzymes which perform deamination and decarboxylation followed by alpha- and beta-elimination [8,11]. Deamination, the first step of the catabolic pathway, removes the amine group from the amino acid, freeing the carbon skeleton. This can be performed on single amino acids, pairs of amino acids (Stickland reaction), or on one amino acid in combination with a non-nitrogenous compound [12]. The products generated by deamination are ammonia and keto-acids [12]. Paired amino acid catabolism, via the Stickland reaction, occurs when one amino acid is decarboxylated and the other is reduced. Alanine, leucine, isoleucine, valine, and histidine are preferentially reduced, while glycine, proline, ornithine, arginine, and tryptophan are preferentially oxidized [13]. Ammonia generated through amino acid catabolism can be used as a nitrogen source for de novo protein synthesis or may be excreted. Keto acids proceed through decarboxylation reactions and can be used to generate short-chain fatty acids (SCFAs) including butyrate, acetate, propionate, lactate, succinate, and formate [13]. Sulfur is also liberated from amino acids during these processes [10]. Not all amino acids are equally suitable for fermentation, and differences in bacterial growth are observed when single amino acids are compared as sole energy sources in culture media. The highest growth is observed from catabolism of glutamate, arginine, glycine, serene, phenylalanine and tyrosine, though tryptophan, aspartate, and alanine can also be used [6]. This complex series of reactions paired with the specific enzyme requirements for catabolizing different amino acids makes predicting the overall metabolite pool in the gut challenging. However, the available information on metabolites generated from the catabolism of specific amino acids may help inform future work focused on understanding what luminal metabolites are generated from proteolytic fermentation when different levels and types of dietary protein are consumed.

## 3. Proteolytic Fermentation Produces Diverse Metabolites

Identification of metabolites generated through proteolytic fermentation in the gut lumen has been limited to date by the complexity of the luminal contents and by limitations in classifying metabolites as host- or microbe-derived [4]. Similar to fiber fermentation, protein fermentation produces short-chain fatty acids; however, these are accompanied by branch-chained fatty acids, ammonia, amines, hydrogen sulfide, phenols, and indoles [14]. Some amino acids have characteristic metabolite profiles, such as those generated for branched-chain and aromatic amino acids, which can be used as an indicator of protein fermentation in the gut [15]. Many of these products are also being identified as bioactive molecules with roles in signaling.

While SCFAs are generated through proteolytic fermentation, total SCFA, acetate, butyrate, and production are lower than what is generated from carbohydrate fermentation, while propionate production remains relatively stable [16]. These differences in SCFA abundance and profile are functionally important as butyrate provides energy to colonocytes, while acetate is thought to have important systemic effects, including in reducing hypertension [3,17]. Butyrate is produced through catabolism of glutamate and lysine by bacterial species, including the potentially harmful *Fusobacterium* spp. [18]. Branched-chain fatty acids (BCFAs) are reliable markers of proteolytic fermentation as they are produced exclusively through the fermentation of branched-chain amino acids. The production of BCFAs increases within 24 h on a high protein diet demonstrating how rapidly dietary protein can alter the host metabolite pool [16,19]. High-protein diets also modify the BCFA profile, increasing the cumulative production of isovalerate, which constitutes a small proportion of the BCFA pool under normal conditions [6,16]. Little is known about the effects of BCFAs on host physiology, but there is some evidence to show that they can be oxidized when butyrate is not available [10]. BCFAs are not thought to be important in colonocyte proliferation or apoptosis *in vitro*. However, a lack of in vivo evidence limits our understanding of what effects an increase in their concentration may have. By their nature, these catabolic processes generate ammonia which can then be utilized in the host urea cycle, or it must be excreted due to its toxicity [10,18]. Ammonia concentrations in the intestine result from the cumulative effects of enterocyte metabolism, microbial deamination, and microbial protein synthesis [6,20]. Increased carbohydrate fermentation and bacterial growth can decrease ammonia concentrations in the gut due to higher incorporation of nitrogen into microbial cells [21].

In addition to BCFAs and ammonia, increasing interest is being paid to other metabolic derivates of proteolytic fermentation. Some of these products are implicated in diseases, including colorectal cancer, while others, such as dietary polyamines, play important roles in small intestine mucosal cell physiology and immune system development [4,22,23]. Polyamines are produced via a diverse set of pathways which result in the decarboxylation of amino acids [24,25]. Many amine producing species from genera including *Bifidobacterium*, *Clostridium*, *Lactobacillus, Escherichia*, and *Klebsiella* have been identified in the gut microbiota [26]. Bacteria utilize polyamines in RNA synthesis, as structural components of cell membranes or peptidoglycan, and to protect against damage from reactive oxygen species or acidic environments [27]. This production of amines during times of physiological stress can result in changes in bacterial pathogenicity, as well as host susceptibility to infection, making these compounds candidates for further exploration with respect to their roles in gastrointestinal infection as well as carcinogenesis which is further discussed in subsequent sections [27].

Ten putrefaction pathways generating these diverse end products have been identified thus far and have been attributed to many of the major microbial phyla in the gut, including Firmicutes, Bacteroidetes, and Proteobacteria [28]. Fermentation of aromatic amino acids may be particularly important biologically. as this generates a wide range of bioactive end products such as phenol and p-cresol (Tyrosine), or indole and skatole (Tryptophan) [4]. Probabilistic pathway construction has identified three microbial pathways catabolizing tryptophan and generating a total of 10 products, six of which participate in host metabolism [4]. Evidence of the role of microbes in the production of these products is demonstrated by their minute abundance in the cecal contents of germ-free mice [4]. Microbiota metabolism of tyrosine can produce phenol, a product which is not detected in the absence of microbes [4]. These metabolites are excellent candidates for further exploration and validation to identify which microbial species may be important in generating specific metabolites in vivo. Currently, little is known about how the changes in the microbiota impact the production of these bioactive molecules.

## 4. Many Microbial Species Contribute to Proteolytic Fermentation

Identifying the bacterial species responsible for proteolytic fermentation has primarily used correlative methods and culture on amino acid containing media. Typically, these experiments have used branched-chain fatty acids as markers of proteolytic fermentation. A culture of digesta obtained from the human colon suggests that isobutyrate forming species may account for up to 40% of the total anaerobes in the intestine, while the abundance of isovalerate producers is more variable [6]. The relative abundance of these species may be altered in high-protein diets, where increased isovalerate production is observed [16]. Species implicated in proteolytic fermentation in vitro include bacteria in the genera *Clostridium*, *Fusobacterium*, *Bacteroides*, *Actinomyces*, *Propionibacterium*, as well as *Peptostreptococci* [6]. Clostridium is important for lysine and proline utilization via fermentation in the colon, while *Peptostreptococci* drive tryptophan and glutamate catabolism [29]. Aromatic amino acid metabolism reactions are thought to be primarily performed by *Enterobacter* and *Escherichia* spp. [4]. BCFA abundance has also been correlated with decreased Firmicutes and increases in unknown Bacteroidetes, as well as *Prevotella* spp., *Bacteroides ovatus*, *Bacteroides thetaiotamicron*, and *Clostridium* spp. in a TIM-2 model of high-protein diets [16]. These changes occurred despite acidic fermentation conditions which likely inhibited some bacterial proteases [6,16]. Another method, examining the presence of putrefaction pathways in gut microbes *in silico*, has also implicated *Bacillus* spp. in protein fermentation, despite not being identified in culture experiments [28].

A newer approach using KEGG pathway analysis of annotated human gut bacterial genomes and probabilistic pathway construction shows that Proteobacteria possess the broadest gene coverage of amino-acid reactions, though only 9% are unique to this phylum [4]. This can be explained by the many metabolic functions that are conserved across species, resulting in high functional redundancy in the microbiome [4,30]. When KEGG classification is performed on all predicted microbial reactions, the largest identified category is amino-acid metabolism (16% of all reactions); however, it is important to consider that approximately 21% of the reactions are unclassified, highlighting just how much work is still required to determine the true functional capacity of the gut microbiome [4]. Given the high number of unculturable species in the gut and the simplicity of single or paired amino acid media, it is likely that not all species contributing to proteolytic fermentation in vivo have been identified. Likewise, the species with the greatest capacity for proteolytic fermentation cannot be identified in a non-competitive environment. Due to the differences in substrate abundance, community membership, and species richness in different locations of the gut, it is important not only to establish which species are participating but also to examine how processes may differ in the small and large intestines [8].

## 5. Proteolytic Fermentation in the Small Intestine Affects Host Amino Acid Balance

Despite the fast transit time and a high degree of host absorption of peptides and amino acids, evidence suggests that microbial utilization of amino acids begins in the small intestine [8,31]. A shift in ileal microbiota structure has been demonstrated in response to dietary protein levels [32]. Compared to a high protein diet (16% crude protein), a moderate dietary protein (13% crude protein) decreases *Clostridiaceae* and biogenic amines in the ileum of pigs while also increasing tight junction proteins claudin and occludin [33]. This contradicts other studies showing a beneficial effect of amines on gut function and suggests that there may be a threshold effect beyond which amine production is detrimental to gut barrier function. Polyamines are readily absorbed from the gut lumen and are important regulators of cellular metabolism, growth, and proliferation [34]. However, at high concentrations amines have been shown to cause inflammation and epithelial shedding of gastrointestinal mucosa, as well as disorganization of pancreatic tissue in young animals [35].

New evidence also shows that 30–50% of essential amino acids may be utilized in first-pass metabolism occurring in the small intestine; microbial utilization is thought to play a role, though the extent of utilization is not known (Figure 1) [20]. This is an important consideration as microbial and endogenous proteins resulting from first-pass metabolism are poorly absorbed once they pass the ileocecal junction [20,36]. Sequential sub-culture experiments demonstrate that *Klebsiella* spp., *Streptococcus* spp., *E. coli*, and *Mitsuokella* spp. from the porcine small intestine utilize amino acids at an appreciable rate of 50–90% over 24 h, which may impact the overall small intestine amino acid metabolism [31]. These microbes preferentially use lysine, threonine, arginine, and glutamine for growth, but also demonstrate uptake of leucine, isoleucine, valine, and histidine [31]. Importantly, this study demonstrated that the availability of specific amino acids could alter the population of intestinal bacteria that were able to grow *in vitro.* For example, *Acidaminococcus fermentans* growth could only be supported by arginine, glutamate, or histidine presence in the media [31]. A limitation of this study is the long incubation times and high concentrations of amino acids compared to what would exist in the intestine. Additionally, the disappearance rather than the metabolic fate of the amino acids in these mixed cultures was observed, and their relative use for energy or protein synthesis was not established. Further experiments have shown that in mixed microbial cultures, overall amino acid utilization is lower than in pure cultures of *E. coli* or *Klebsiella* spp. alone [37]. When the fate of these amino acids was examined in more detail, mixed cultures of ileal bacteria showed large amounts of lysine catabolism when compared to incorporation into the microbial protein [37].

Threonine is also highly oxidized by *Klebsiella* sp. and *E. coli* compared to mixed cultures, which is an interesting difference compared to the overall microbial community which preferentially oxidize glutamine and arginine [37]. This suggests that in pathological conditions where *E. coli* or *Klebsiella* are highly abundant in the gut, amino acids may become less available to the host, while protein fermentation by-products may contribute to disease pathology through increased inflammation. These findings also demonstrate that amino acid utilization by small intestinal microbes may be an important consideration. However, our current understanding of the physiological relevance is limited, as it is unclear what percentage of required amino acids are sequestered as part of the normal intestinal microbiota function. Furthermore, the culture conditions may not completely reflect the community in vivo. Future work feeding N^15^ labeled amino acids to animals colonized with defined communities of microbes (e.g., *E. coli* free or colonized [38]), could help to further elucidate how these findings translate in the complex gut environment. However, nitrogen recycling is a major limitation to the use of labeled amino acids. Linking proteolytic end products to specific microbes in defined communities may be a more robust way to identify markers of the proteolytic pathway. Once markers of proteolytic fermentation are well established, this could also then be evaluated in humans.

## 6. Proteolytic Fermentation in the Large Intestine Generates Bioactive End-Products

In humans, carbohydrate fermentation occurs primarily in the proximal colon, resulting in a distal colon containing low fermentable substrate. Protein flowing into the large intestine may come from undigested food, bacterial cells, and endogenous gut losses. These endogenous losses include protein from enzymes, mucus, and sloughed epithelial cells [39]. The small intestine makes the largest contribution to endogenous losses and can be affected by factors such as protein quality and fiber intake [39]. Fiber type also affects the degree of endogenous losses. Wheat bran, for example, increases endogenous loss, while no effect is seen with cellulose [40]. This effect may depend on the degree to which a given fiber type increases enzyme secretion and mucous production or decreases the digestibility of amino acids in the small intestine [39,41]. The resulting flow of protein into the large intestine may not result in detrimental accumulation of end products in these cases, as it is accompanied by microbial accessible carbohydrates.

When carbohydrates are readily available, proteolytic fermentation is limited to the distal colon, where carbohydrates are depleted, creating an energy deficient environment and a pH closer to neutral [5,6]. When protein reaches the distal colon, the slow transit time and limited host absorption facilitate intense microbial proteolysis and accumulation of metabolic end products [10,36,42]. Acidic pH can greatly reduce the production of BCFAs, while in the presence of some carbohydrate more isobutyrate and isocaproate are produced [6]. In the colon, Clostridia have been identified as particularly effective at amino acid deamination via the Stickland reaction [6,11]. Through this reaction, *Clostridium* spp. are the major fermenters of lysine and proline, while the *Peptostreptococcus* spp. utilize glutamate and tryptophan [29]. Preferential uptake of amino acids can be observed through the free amino acid pool where differential abundance is observed, suggesting that some amino acids are not utilized (hydroxyproline and taurine), while others may have different fates depending on the location in the colon [6]. These alterations in free amino acid availability may have less of an impact in the colon due to the high degree of microbial utilization [18]. The effects of colonic protein fermentation on host health may therefore be considered to be based on the accumulation of bioactive metabolic products rather than changes in amino acid abundance.

## 7. Diet Affects Proteolytic Fermentation

While the metabolic pathways and microbial species involved in protein fermentation have been described in culture and modeling experiments, how these processes function under different dietary patterns still largely remains to be determined. Temporal changes in substrate availability, host absorption, and production of glycoproteins could all affect the overall degree of protein fermentation, making methodical dietary studies necessary. Certainly, it has been demonstrated that the effects of high protein diets occur quickly, with rapid changes to the microbiota and metabolites observed within 24 h in both model systems and human trials [16,19]. Not only does high protein intake need to be considered, but the low dietary fiber intake of most Western diets may complicate dietary studies due to the altered abundance of fiber fermenting species in the gut [5]. Low fermentable carbohydrate intake results in low substrate availability and in turn a higher pH. This change may cause the length of the colon to more closely resemble the normal conditions of the distal colon [43]. Bacterial proteases work best at neutral pH, and are thought to be inhibited by SCFA production. Therefore, the relationship between dietary fiber and dietary protein may result in changes to fermentation location [23]. This is further supported by experiments comparing different types of dietary fiber, which suggest that fiber type is important in altering the relative production of branch-chained fatty acids through effects on the fermentation location within the colon [44]. These results are somewhat inconsistent where other high-protein, low-carbohydrate diets have shown an overall decrease in SCFA production without an increased relative abundance of BCFAs [45]. This could have been due to the loss of overall species richness observed in the same study [45]. High levels of dietary protein from animal sources also selects organisms that are more bile resistant, a function of concurrently increased fat intake [19]. Increasing bile tolerant organisms and decreasing the abundance of fiber fermenting species suggests that a close examination of compartment-specific effects may reveal shifts in fermentation dynamics throughout the gut.

The suppression of protein fermentation by dietary fiber is thought to be due to the decreasing demand for amino acids as an energy source, and lower pH from SCFA production inhibiting proteolytic enzymes [6]. These effects result in a reduction in the amount of potentially undesirable metabolites formed [46]. Carbohydrate presence alters amino acid utilization by microbes, reducing the uptake of some amino acids, such as tyrosine, and increasing the use of others, including valine [6]. Carbohydrate fermentation can also strongly inhibit the formation of specific products such as p-cresol, which is further described below, even when the degradation of their amino acid precursor (tyrosine) is still occurring [47]. This may be specific to aromatic amino acids, as their complexity lends itself to a wider range of degradation pathways and metabolic end products [4]. The importance of this dietary context is demonstrated in a study comparing diet and fecal microbiota in African-Americans, a population with a high incidence of colorectal cancer (CRC), to rural South Africans, a population with a low incidence of CRC. Higher levels of proteolytic fermentation products were observed in rural South Africans. However, this was observed alongside increased carbohydrate fermentation and a lower incidence of colorectal polyps [48].

**Figure 2:**
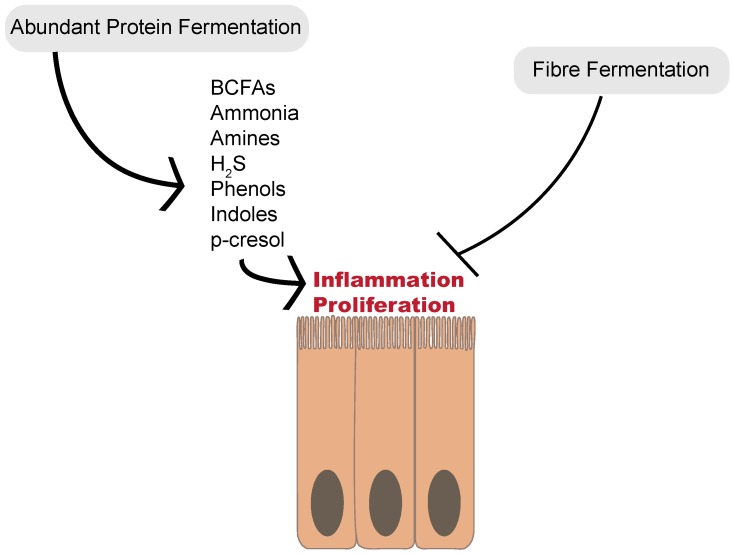
Abundant proteolytic fermentation generates a multitude of compounds that may cause the inflammation and proliferation of colonocytes, and in turn, colorectal cancer. Increased fiber fermentation and short-chain fatty acid production appears to be protective against colorectal polyp development, even when protein fermentation products are abundant.

This finding of increased proteolytic fermentation despite lower CRC incidence suggests that the increased carbohydrate fermentation observed in rural South Africans may exert a protective effect against the effects of proteolytic metabolites on CRC development. When metabolic networks are examined, branched-chain amino acid fermentation appear to be increased in the African diet. However, urinary *p*-cresol is higher in African-Americans before dietary intervention [48]. This supports the suggestion that high fiber intake may alter protein fermentation pathways and provide protective effects against inflammation and disruption of cell cycles (Figure 2). The mechanisms underlying this shift in proteolytic fermentation away from deleterious metabolites remain to be elucidated. Possible mechanisms could include the inhibition of particular fermentation pathways or fermentation by specific microbial species. Likewise, fiber fermentation and resulting changes in digesta viscosity could alter the interactions between metabolites and the mucosa limiting these metabolites’ detrimental effects. More information on these complex metabolic interactions is needed before strong recommendations can be made.

## 8. Protein Fermentation is an Important Consideration for Host Health

The health effects of increased protein fermentation are not entirely clear, but high protein, low carbohydrate diets for weight loss have been shown to increase the proportions of phenylacetic acid from phenylalanine degradation and *N*-nitroso compounds, raising questions about the long-term effects of these diets on colonic health [46]. In athletes, protein supplements have been shown to alter the composition of the microbiota, increasing the abundance of Bacteroidetes while decreasing *Rosburia*, *Blautia*, and *Bifidobacterium* [49]. However, no metabolites of microbial proteolytic fermentation were measured, and while some *Bacteroides* species can ferment protein, more information is needed to ascertain whether these supplements affect microbial metabolism. Another consideration is that the benefits of butyrate may also be decreased if it is produced through protein fermentation. This is due to the inflammation generated by the release of ammonia, which decreases butyrate transporter expression, and in turn, butyrate uptake by colonocytes [50]. Increased ammonia also decreases colonocyte oxidation of butyrate which is replaced by increased glycolysis [51]. These changes can decrease intestinal cell integrity and barrier function [10,18].

A comparative analysis of colorectal cancer patients and healthy controls found a relative enrichment of species capable of fermenting protein. Putrescine and histidine pathways were most common, and *Fusobacterium* was identified as an important differentially-abundant genus [28]. This supports previous studies reporting an enrichment of *Fusobacterium* in colorectal carcinoma [52]. *Fusobacterium nucleatum* adheres to colonocytes and generates an inflammatory host response [53]. Hydrogen sulfide and ammonia are also generated by *F. nucleatum* during the degradation of cysteine and production of butyrate, respectively [18,54]. Sustained exposure of colonocytes to free ammonia generated during proteolytic fermentation may contribute to the development of colorectal carcinoma (CRC) [55]. Like ammonia, *p*-cresol may also cause DNA damage and alter the cell cycle, decreasing colonocyte proliferation; this may be due to its effect on colonocyte oxidative metabolism and ATP production [56,57]. This decrease in cell viability and the disruption of the cell cycle have also been demonstrated in atherosclerosis, alongside an increase in reactive oxygen species production [58]. Urinary *p*-cresol is also implicated in kidney disease through its effects on endothelial cells [59]. These findings suggest that *p*-cresol is an important metabolite to consider when the detrimental effects of proteolytic fermentation are considered locally and systemically on colonocytes. However, not all proteolytic fermentation products are associated with increased CRC risk; recent studies suggest a protective effect of dietary polyamines against CRC development [60]. Thermolyzed protein was not shown to promote colon cancer in short-term models [61]. This underscores the importance of further examination of specific metabolic pathways and products in the context of long-term dietary patterns, including consideration of dietary fiber intake.

**Figure 3:**
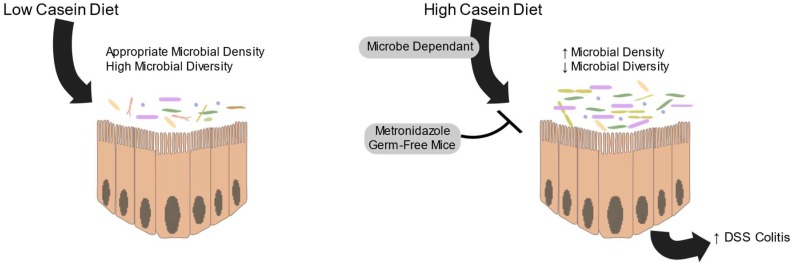
High casein diets cause an increase in microbiota density and a decrease in microbial diversity. This change in the microbiota results in an increase in DSS colitis severity. High casein diets do not have this effect in germ-free mice or if microbial density is controlled using metronidazole.

Protein intake is also associated with increased severity of DSS induced colitis, an effect that is not seen in germ-free or antibiotic-treated mice (Figure 3) [62]. High levels of fermentable protein decrease the expression of claudins in both the distal and proximal colon, which may compensate for the detrimental effects of these metabolites [63]. Highly fermentable protein has also been demonstrated to increase the expression of inflammatory cytokines in the mucosa, even if the microbial composition is not different [64]. These detrimental effects of proteolytic fermentation are conflicted by other studies, as reviewed by Sridharan et al., (2014) [4], which show that indole derived from tryptophan deamination decreases intestinal epithelial inflammation and improves barrier function via tight-junctions. These local effects may be important in pathogen resistance and colorectal cancer, though much more work is needed.

Metabolites generated through proteolytic fermentation also enter systemic circulation and travel to the liver and peripheral tissues, exerting wider effects [42]. In a twin study, metatranscriptome analysis of the microbiota shows decreased expression of genes associated with amino acid degradation pathways in microbiota from obese individuals compared to their corresponding lean twins [65]. This decreased capacity for degradation could lead to an increase of these amino acids in systemic circulation, particularly if high levels of protein are consumed and could be detrimental for health. These changes in amino acids parallel the increase in circulating branched-chain and aromatic amino acids seen in type-2 diabetes mellitus (T2DM) and insulin resistance, suggesting that altered fermentation may be important in T2DM [66]. Examination of plasma metabolites in a well-characterized population of obese adolescents showed a strong correlation between higher plasma levels of BCAAs, tryptophan, lysine, and glutamate and non-alcoholic fatty liver disease [67].

In the same study, baseline plasma valine was identified as predictive of liver fat accumulation over the following two years [67]. Though not completely understood, this is suggestive of changes in intestinal permeability or microbial amino acid synthesis, which may occur as a result of altered microbial populations seen in T2DM and metabolic disease. Another factor that should not be overlooked is the de novo synthesis of amino acids. When adults consume a protein-adequate diet, microbially-produced amino acids have been shown to be a significant contributor to the plasma amino acid pool [68]. While some absorption of amino acids may be possible in the large intestine, there is a limited body of evidence to date to suggest that this absorption is physiologically relevant [36]. More work is needed to determine the relative amounts of microbially-produced amino acids reaching systemic circulation from the large or small intestine and how this may be altered in high protein, low fermentable carbohydrate diets.

The role of proteolytic fermentation in health goes beyond metabolic disease, as metabolites of aromatic amino acid metabolism are capable of binding the aryl hydrocarbon receptor (AhR). This is an important transcription factor which may implicate them in endocrine regulation, cytokine signaling, and, unlike free ammonia or *p*-cresol, they may decrease cancer development [4,69]. Indole in particular may have important effects on the nervous system and may enhance neurodevelopmental or psychiatric diseases [70,71]. In cases of a moderate and chronic overproduction of indole by the gut microbiota, rats display increased anxiety-like behavior [70]. Indole has also been shown to be protective in mouse models of multiple sclerosis, further demonstrating its importance in connecting the gut microbiota to health outcomes in the central nervous system [72]. Indole is only produced when tryptophan is in its free amino acid form, highlighting the need for consideration of how the overall diet and microbiome affect the production of important bioactive molecules from dietary protein.

## 9. Discussion

Proteolytic fermentation is a highly networked process that can exert many effects on the host. The changes in proteolytic fermentation based on fiber availability suggest that examining the role of protein fermentation on health must also consider the carbohydrate requirement of the gut microbiota [5]. While different effects of carbohydrate type on proteolytic fermentation have been observed [44], more metabolite-level information from the proximal colon and small intestine is necessary to truly understand the significance of these differences. Measurements of fecal components include a large contribution of bacterial secretions and cellular components and therefore may not represent the changes occurring further up in the gut [6]. Untargeted mass spectrometry methods can identify compounds with increasing specificity and efficiency; however, understanding the health implications of the products detected is limited. There is a need for more metabolome data from different gut compartments in healthy vs. disease states, similar to the work done on Crohn’s Disease [73]. Likewise, predictive studies are limited by the accuracy of KEGG databases where ambiguous or missed pathway assignment can interfere with the accuracy of prediction [4]. This highlights the need for further studies to elucidate community-wide changes in microbiota and metabolites associated with proteolysis and to further determine which species are implicated, as well as how these networks come together at the level of the whole community.

The work described previously suggests many avenues to further explore the role of proteolytic fermentation on health. For example, the differences in amino acid utilization between some opportunistic pathogens (*E. coli* and *Klebsiella* sp.) in the small intestine provides an interesting avenue for exploration of how the dietary amino acid profile may be an important consideration in disease states. To date, there is no consensus model of gut microbiota metabolism, and experiments trying to identify microbial proteolytic products have used germ-free mice for comparison [4]. This approach does not allow for the evaluation of which pathways are favored in a colonized gut. There is a need to pair metabolic pathway predictions on individual species and communities with validation experiments that discriminate products of microbial metabolism or shared host-microbial metabolism from host products, as not all of the predicted pathways may occur in the reality of the gut environment. For example, host-only predicted pathways for aromatic amino acid degradation were not detected upon validation in a germ-free model, suggesting that these pathways are not favored in the complex host-microbe metabolic network [4]. A search of the literature also revealed no studies examining the potential toxicity of protein metabolites to other microbes in vivo. Given the ability of other microbial products to limit the growth of community members, it seems plausible that some species may be negatively affected by the presence of proteolytic fermentation products. This could mean that alterations in microbial species abundance are due not only to changes in available niches but also due to species loss from toxic products.

Considering the intricate interactions within the microbial community and with the host, more information is needed that explores beyond microbiota composition and cross-sectional studies to examine specific changes in microbial species and metabolites when dietary proteolytic fermentation is occurring. This missing information will strengthen our understanding of the mechanisms by which dietary change alters the functional profile of the microbiome and in turn affects host health and metabolism.
